# Why Intelligence Is Missing from American Education Policy and Practice, and What Can Be Done About It

**DOI:** 10.3390/jintelligence8010002

**Published:** 2020-01-03

**Authors:** Robert Maranto, Jonathan Wai

**Affiliations:** 1 Department of Education Reform, University of Arkansas, Fayetteville, AR 72701, USA; 2 Department of Psychology, University of Arkansas, Fayetteville, AR 72701, USA

**Keywords:** education policy, teacher quality, intelligence in education, educational leadership, education history

## Abstract

To understand why education as a field has not incorporated intelligence, we must consider the field’s history and culture. Accordingly, in this cross-disciplinary collaboration between a political scientist who studies institutions and a psychologist who studies intelligence, we outline how the roots of contemporary American Educational Leadership as a field determine its contemporary avoidance of the concept of intelligence. Rooted in early 20th century progressivism and scientific management, Educational Leadership theory envisions professionally run schools as “Taylorist” factories with teaching and leadership largely standardized, prioritizing compliance over cognitive ability among educators. Further, the roots of modern education theory do not see the intelligence of students as largely malleable. Hence, prioritizing intelligence is viewed as elitist. For more than a century, these assumptions have impacted recruitment into education as a profession. We conclude with ideas about how to bring intelligence into mainstream schooling, within the existing K-12 education institutional context. We believe that better integration of intelligence and broader individual differences research in education policy and practice would lead to more rapid advances to finding evidence based solutions to help children.

## 1. Introduction

This essay is a collaboration between a political scientist and a psychologist who each research areas related to U.S. education policy. The political scientist (Maranto) has served in the U.S. Government and a local school board and has conducted education policy research for decades. The psychologist (Wai) started out as a researcher in the field of intelligence, and now studies education policy through the lens of psychology, including how and where the concept of intelligence can be more fruitfully integrated into education policy research and practice.

Our primary goal in this article is to explore why the notion of intelligence is largely absent in the field of education, consider why the notion of intelligence could be useful in education, and propose some ideas about what can be done. We hope to guide future work and offer intelligence researchers a better understanding of the cultural and attitudinal barriers to incorporating intelligence research into the routine policies governing the practices of American public schools. Our proposed solutions come both from the perspective of intelligence researchers hoping to impact education research and policy but also from the perspective of how to influence researchers and others who work in education and schools.

## 2. Why Might the Notion of Intelligence Be Useful in the Field of Education?

*Integrating established information from one field into another*. The notion of *g* is among the most established, replicated, and influential predictors of numerous life outcomes, including educational performance and attainment (Gottfredson 2004; Jensen 1998). Therefore, acknowledging and accepting individual differences in intellectual capacities and learning rates among students can both lead to a more robust and coherent research literature in the broad field of education research (for some concrete examples of how intelligence can help education and its research, see Wai et al. 2018) as well as finding solutions that will work, and ruling out those unlikely to help children. For example, understanding that intelligence or g is measured in pretty much any standardized ability or achievement test (by proxy; e.g., Carroll 1997; Frey and Detterman 2004; Koenig et al. 2008) suggests that literatures could be productively informed by one another just by this link alone. How would this reorient our thinking about the literature in education broadly? As Gottfredson (2004, p. 45) correctly notes: “By embracing rather than rejecting the scientific knowledge about *g*, educators can develop curricula and classroom techniques that well serve the nation’s cognitively diverse students.” The investment of *g*, after all, is key to the acquisition of content knowledge, and unfortunately, as we document in the next section, the field of education has little interest in intelligence research and considerable skepticism regarding content knowledge. Academic fields are often siloed, communicating mainly to one another. The body of research from the field of intelligence would greatly improve the research literature and rate of solution finding to improve the education and life outcomes of students. It could also improve education policy by helping suggest which areas of policy are likely to productively help students, and which areas probably will not (e.g., Asbury and Wai 2019). Accounting for the intelligence of students could positively affect the educational practices that impact students, teachers, principals, and school systems. Of course, intelligence researchers can also productively learn from the field of education, and we stress that interdisciplinary integration requires information crossing boundaries in all directions.

*Accounting for intelligence among all groups of people in schools*. Beyond integrating the information about the intelligence of students into education research and education policy, accounting for the intelligence of teachers, administrators, or other people who interact with students in schools may also be useful. For example, a large body of research literature from the field of industrial/organizational (I/O) psychology illustrates that *g* is enormously important for job performance (e.g., Schmidt and Hunter 1998, 2004). Research has indicated that individuals who choose to become teachers in the U.S. also tend to be drawn from the lower end of the ability distribution, at least among college graduates (e.g., College Board 2014; Wai et al. 2009; Wolfle and Oxtoby 1952). This trend has remained consistent across the last several decades as demonstrated using different ability and achievement measures and different samples over time (Wai 2015). Drawing from the large I/O psychology literature suggesting that *g* matters for job performance, perhaps a better understanding of how *g* matters for the performance of teachers and teaching effectiveness could be a worthwhile avenue of pursuit for education research and policy. We might hypothesize that all else equal, more highly able teachers, for example, might more effectively challenge students, particularly more intellectually advanced or gifted students. Such teachers might also innovate in ways better serving less intellectually advanced students, and those disadvantaged by class or race; indeed, the literature on so called “no-excuses” schools shows that high ability teachers developed and largely staff these schooling models (Thernstrom and Thernstrom 2003).

## 3. How Education’s History Led to the Neglect of Intelligence

In American public schooling, the profession of Educational Leadership dominates both the daily routines of schooling, and educational policy-making, and in these respects the field has far more influence over education than do more systematic education subfields like Educational Psychology, which takes the study of intelligence more seriously. Principals and school superintendents, typically with graduate degrees in Educational Leadership, have considerable influence in state legislatures and in state departments of education, and thus over the certification of public school teachers and leaders. Likewise, former superintendents influence and not infrequently lead schools of education within the mainly second tier universities which train the vast majority of American teachers (Lucas 1999). On a day to day basis, principals and superintendents select and retain (or occasionally, dismiss) teachers, and select which teachers gain promotion to administration. Accordingly, to understand the truncated role of intelligence in American schooling, one must understand Educational Leadership as a profession. Contrasting higher education (until recently) and private schools, American traditional public schools have very hierarchical structures, with slow promotion. As Chubb and Moe (1990) find, typical public high school principals have not taught a course in over a decade, while private school principals (like college presidents) sometimes still teach, and often return full time to the classroom. Through both their training in Educational Leadership programs and via selection and self-selection, as permanent administrators, public school leaders value compliance, teamwork and order (Ingersoll 2003), as indeed have education policymakers since the early 20th Century (Mehta 2013). 

Professions are most malleable in their beginnings, when founders and founding institutions including the educational institutions which train and vet new professionals develop ideologies regarding the profession’s role in society, and its means for realizing those roles. Path development approaches suggest that once a profession institutionalizes, further changes tend to be incremental, with new entrants selected in part for their support of existing institutional norms, cultures, and practices (Klyza 1996; Mehta and Teles 2014). The field of Educational Leadership arose in the early 20th century, and it remains influenced by the doctrines of that time. As classic treatments of the development of Educational Leadership by Labaree (2003, 2004), Callahan (1962), and Rousmaniere (2013) show, the founders of the field lacked optimism regarding the importance of intelligence and its malleability. They conceptualized intelligence as largely predetermined; thus, efforts to improve intelligence through schooling were seen as both quixotic and elitist. While schools could sort students based on their intelligence, schools could not develop intelligence. This influences how modern schools operate on a daily basis, even today (e.g., Burkett 2001; Powell et al. 1985).

Further, in part to distinguish themselves from other university based disciplines and to appeal to pragmatic school boards largely made up of businessmen, the founders of Educational Leadership adopted scientific management models imported from business. Scientific Management increased manufacturing efficiency and compared to Social Darwinism, which influenced prior management philosophies, it marked a significant evolution in a humanitarian direction. Yet it had muted regard for intelligence. As devised by Frederick Taylor and others, scientific management dictated that good managers observe the workplace, break down each step in a production process to find the most efficient techniques, then use bonuses (or threats) to persuade workers to learn and use that “one best way.” Scientific management assumes that all reasonable people agree on goals (more efficient production); thus, it demands managers (in the case of schools, principals and superintendents) with certain technical skills, but no need for the intellectual depth to understand that in education, not all capable people of good will agree on either the goals of schooling, nor the best means to achieve those goals. This management philosophy does not demand intelligence in managers, and positively scorns intelligence in employees. Ideal teachers should be trainable towards compliance rather than out of the box thinkers (Perrow 1979; as regards schooling see Ingersoll 2003; Maranto 2001).

As Callahan (1962) and Rousmaniere (2013), in particular, show, early 20th century Progressive reforms in line with the scientific management administrative doctrines of the day developed large, differentiated public schools intended to function as factories in which male superiors (educational leaders) with the proper graduate Educational Leadership credentials managed largely female subordinates, a matter treated further below. Maranto et al. (2018) found that such cultural norms and personnel practices still have influence in public schooling. Further, as Callahan (1962) documents regarding the early 20th century and Levine (2006) demonstrates in the 21st century, doctoral dissertations in the field of Educational Leadership lack intellectual rigor relative to other areas. Many focus on such matters as the most efficacious bell schedules and the most efficient ratio of custodians to floor space. Indeed, as Ellwood Cubberley, dean of the school of education at Stanford University and one of the founders of Educational Leadership as a field, explained, before 1900 schools had been “a manufacturing establishment running at a low grade of efficiency.” The field of Educational Leadership aimed to change that (see also Labaree 2003, 2004; Mehta 2013; Tyack 1974; Rousmaniere 2013). As Mehta (2013) and Labaree (2004) show, in the early 20th century, many decades before *No Child Left Behind* forced public schools to report test scores to the public, large school systems run by administrative progressives used widespread quantification in efforts to centralize control at the principal and even superintendent level, minimizing teacher autonomy and initiative. Yet, in practice, given the hundreds of decisions teachers must make daily, centralized efforts to manage their complex work are counterproductive. Moreover, scientific management-like efforts to control teachers led their unions through the 20th century to behave more like traditional industrial unions than professional organizations (Ingersoll 2003; Mehta 2013). This is precisely the opposite of how the most successful schools and school systems operate (Ouchi 2009). 

As Labaree (2004) illustrates, *pedagogical progressives* like Dewey influenced schools of education and thus the training and selection of teachers; over time they also watered down course content. Yet the *administrative progressives* who dominated Educational Leadership had far more real world influence. Neither group, as it happens, had enormous regard for intelligence, nor for academic content unless narrowly tailored to utilitarian and typically vocational ends. Further, while in the late 19th and early 20th centuries most principals and many superintendents were women, a key aspect of professionalizing education at a time when professional meant male was putting men in charge. In part to attract men to the field, public education developed athletics and the new field of athletic coaching; thus men could coach for a few years before becoming secondary principals and eventually superintendents. Even today, while most teachers are women, most educational administrators are men. Nationally, 53% of male principals and a higher percentage of superintendents are former coaches. Interestingly, those who select into the field tend to be relatively conservative, comfortable with predictable employment, and even traditional gender roles (Maranto et al. 2018; see also Rousmaniere 2013). Generally speaking, for both educational administrators and school board members, athletic competitions are centers of school life while academic competitions and day to day teaching are largely invisible (Maeroff 2010, p. 18).

One could imagine an alternative past in which Educational Leadership as a field of study embraced relatively rigorous subfields of Psychology such as Cognitive or I/O Psychology, or perhaps even History and Government. These are relatively higher intelligence academic professions, on average, than Education, though all remain lower than the hard STEM disciplines and have so for decades (Wai 2015; Wai et al. 2009). Indeed Maranto et al. (2010) call for this remaking of the schools of education which train public school teachers and leaders, copying the successful reforms of business schools in the 1960s, which reorganized their requirements around three rigorous academic disciplines: I/O psychology, statistics, and economics. Such reforms did not, and likely could not occur in Educational Leadership, nor in education generally. The academic field of business within higher education cannot force private businesses to hire only managers with MBAs. In contrast, state departments of education, influenced by schools of education, have required that the vast majority of traditional public schools hire the teachers and administrators schools of education have trained and certified; thus, limiting the impetus for reform. As Hirsch (1996) and Stotsky (2015) detail, in this way K-12 public education has, like most professions, developed distinct selection and training pathways, organizations, certification systems (enforced by law), and specialized terminology. Stotsky notes that at times in the past, in some states, teacher certification requirements had significant rigor via teacher licensure tests. Unfortunately, since at least the 1950s, states have lowered standards to make sure public schools can recruit sufficient numbers of teachers to fill classrooms.

As Koedel (2011) and others have demonstrated, as a college major Education on both undergraduate and graduate levels lacks rigor; thus Moe (2011) portrays the field as having “phony professionalism” with positions based on non-challenging undergraduate, masters, and doctoral degrees rather than actual knowledge or achievement. Such certifications (and confusing terminology) lend a certain legitimacy, drawing support from teachers unions, school boards, and of course the schools of education that provide them. This role lacks credibility among academic elites, however. As Labaree (2004, p. 91) tartly observed:

“Those teaching in the university think of those in ed schools as being academically weak and narrowly vocational. They see ed school teachers not as peers in the world of higher education but as an embarrassment, who should not be part of the university at all. To them the ed school looks less like a school of medicine than a school of cosmetology.”

Elites increasingly attend and typically send their own children to elite private schools that do not use teacher or leader certification as criteria for employment. As Maranto and McShane (2012, p. 61) document, most recent presidents received their education in and sent their own children to such private schools, indicating their distrust of existing systems of education licensure and professionalism. Further, very few doctorates of education become college or university presidents: Among the eight Ivy League universities and eight “Public Ivies”, none is currently led by an Ed.D. 

Generally, with the rise of mass schooling, educational administrators and school boards fashioned schools to focus on non-academic foci, since they assumed few students could handle serious academic work, which itself had little or no value unless it could be connected directly to vocation. Accordingly, schools would operate to provide custodial care for students to keep them off the streets and the job market, and to socialize them into eventual factory work. Historically, Labaree (2003, 2004) and Maranto and McShane (2012) consider the *Cardinal Principals of Secondary Education* issued in 1918 by the U.S. Government and the National Education Association (then a school administrator’s organization) to be an important foundational document of American secondary education, enumerating and justifying the key objectives of schooling at a time when secondary schooling expanded rapidly. As noted, rapid growth meant that new organizational ideologies and operational practices could be imposed and institutionalized among new personnel and in new schools. Importantly, and in sharp contrast to prior justifications for secondary schooling, only one of the seven *Cardinal Principles* concerned academic achievement. This single goal, *command of fundamental processes*, was to cover nearly all scholarly disciplines. (The other goals are health, worthy use of leisure, citizenship, worthy home membership, vocation, and ethical character.) While few living teachers and educational leaders have ever heard of the *Cardinal Principles*, the document still defines the field. Indeed, nearly 60 years after the *Principles*’ publication, a superintendent told one of us (Maranto) “you do not understand the purpose of the public education system. The purpose of public education is *not* to educate students. The purpose of public education is to provide an education for those *few* who want it.” Other students would have to be kept in schools since otherwise “crime would go up, unemployment would go up” (quoted in Maranto and McShane 2012, p. 11). 

If the key goals of schools are sports and other extracurricular activities to keep students in custodial settings rather than traditional intellectual goals, then there is no particular need for schools to stress intelligence, or even recognize it as a concept; indeed this may well be seen as elitist, particularly at a time when schooling expanded rapidly. As Labaree (2004, p. 109) observes:

“If school subjects have to be adjusted to the capacities of the students and to the requirements of the job market, and if most students have modest capacities and most jobs have modest skill requirements, then only a few classes need provide a rigorous academic content for the college bound elite, while most students need classes that are less academic, less demanding, and better suited to their modest future roles in society. This is a straightforward prescription for diluting academic content.”

Though this area is under-studied, evidence suggests that in hiring teachers, typical public school principals do not take cognitive ability nor subject matter knowledge into account (Shuls and Trivitt 2015; Kramer 1991). In short, the ideology of education as a field largely explains its failure to embrace intelligence as a goal, or even as a concept. Further, until other professional occupations were open to women and minorities, high levels of female and minority teacher (though not white male administrator) human capital were assured. Only since 1970 has teacher quality (as measured by cognitive ability measures like the SAT) fallen. Estimates indicate that among female high school graduates in the top tenth of cognitive ability, the proportion entering teaching declined from roughly a quarter in the early 1970s to roughly a tenth in the early 2000s (Gastic 2014; Maranto 2015). This happened at the very time when elites began to stress the importance of students making gains in cognitive ability for national economic success. Yet the relatively low level of teacher cognitive ability that has stretched across many decades back to the 1940s (see Wai 2015 for testing data across 5 independent sources) has itself played into the approaches of traditional education leaders, teachers unions, and many educational reformers. To varying degrees, all are stuck in the early 20th century factory models of schooling, and low cognitive ability teachers may be more compliant with regular and largely symbolic reforms developed by policy-makers and implemented by educational leaders; they will also offer more solidarity to unions (Mehta 2013; Moe 2011). Once a field gains a reputation for low cognitive ability, it attracts new entrants who do not value intelligence, starting with who chooses education as a college major (Armstrong and Hamilton 2013; Stotsky 2015; Wai 2015). Further, school leaders rarely terminate teachers for poor teaching (Moe 2011) and likely as a result, the field attracts the risk averse (Bowen et al. 2015).

Stotsky (2015) argues that, in part, reducing the rigor of teacher certification tests like the Praxis reflects the desire for a racially diverse teaching core. We argue that this was not really a factor until the 1970’s, long after the institutionalization of state level certification practices. Diversity goals did not lower barriers to entry into the teaching profession but were later used by education professors to justify the reduced academic rigor. Ironically, the existing teacher workforce is overwhelmingly non-diverse, so if low barriers to entry are meant to facilitate a diverse workforce, they are failing to achieve this goal (Maranto et al. 2018).

Defining itself as distinct from and more pragmatic than the more scholarly segments of higher education, Education as a field values the trappings of education, non-rigorous degrees and certifications for employment—but not actual reflections of intelligence or content knowledge. Hirsch (1996) recounts how conventional educators often expressed astonishment that he actually enjoyed learning the facts and stories that went into his engaging Core Knowledge curricula. One contemptuous educator asked if Hirsch actually thought that learning more content made him a better person (Hirsch 1996, p. 55). Decades before “fake news,” the field of Education had espoused limited appreciation for facts, instead embracing the approach that “we teach children, not subjects.” To be clear, academic skill development and subject matter knowledge should not be the sole goals of schooling. Personal relationships including school level affinities (“school spirit”) should also matter. Yet as Maranto and McShane (2012) document, over the past three decades of U.S. education reform, elites have consistently sought to make U.S. schooling more academic and less social and thus more oriented toward academic skill development, to resemble schooling in most European and Asian systems. The field of Educational Leadership, charged with implementing elite driven reforms, by and large does not support those reforms.

This does not mean that intelligence is entirely absent from schooling. If schools could not celebrate and develop intelligence, which was thought to be largely inherent and not terribly useful to vocation, schools could sort out the smarter students from the rest. In fact, according to Tyack and Cuban (1995, p. 58), in the 1920s “hype about the virtues of intelligence tests and tracking of students into differentiated courses of study led to rapid adoption of these practices.” Today this has largely fallen out of favor in educational theory, yet in actual schools academic tracking is alive and well, and arguably serves legitimate educational purposes despite its ostensible unpopularity (Loveless 1999). Thus, education reformers 100 years ago took intelligence tests heavily into account for social efficiency reasons. These policies and practices have fallen out of favor today in the academy and among education policymakers but retain a quiet but widespread presence in actual schools in matters like tracking, entry into gifted and talented and advanced placement programs, entry in magnet schools, and college advisement. Moreover, it is true that within public schools there is considerable competition over academic awards such as National Merit Scholarships, though in practice educational administrators tend to see these as a function of student privilege (Demerath 2009). 

## 4. Why Is the Notion of Intelligence Absent in the Field of Education?

The brief journey through education history was hopefully useful in understanding the muted and often absent role of intelligence in education and education reform. Roughly a century ago there was broad acceptance of intelligence tests and selecting students for matching them with differentiated curricula (Tyack and Cuban 1995), but that is less true today. Although it is true that there are world class scholars doing intelligence-research in educational science in the US, intelligence as a topic in US education remains less widely accepted, especially in relation to the strength of the evidence the field has produced. Additionally, the field of education, as also illustrated through history, is not really about evidence. As intelligence researchers and as social scientists, our goal is to uncover what is true scientifically. That is the not the primary goal of the field of K-12 education, as we described earlier (Hirsch 1996; Ravitch 2000).

Controversies surrounding the publication of Jensen’s (1969) “How much can we boost IQ and scholastic achievement?” and Herrnstein and Murray’s (1996) *The Bell Curve* have also contributed not only to the absence of the open discussion of intelligence in education, but more broadly among the American public and especially in academia. Finally, academic subfields, such as Educational Leadership and the field of intelligence tend to act as silos of information, since academic incentive structures, conferences, journals, and other niche factors tend to reduce the motivation for scholars to integrate information across various subfields. More broadly, there appears to be largely segregated scientific groups and literature bases with one group doing intelligence research and another group neglecting intelligence, with either group not really noticing each other much. One exception might be educational psychology where some scholars have worked to show the relevance of intelligence for education. There is a tension between scientific knowledge about intelligence and a substantial neglect of this knowledge in the daily education work in schools. There is an additional tension between the large neglect of intelligence by teachers, administrators, and other stakeholders and on the other side the daily experience of teachers while teaching that reveal that students differ in their intellectual capacity to learn. Intelligence scholars are unlikely to read education research and education researchers are unlikely to read intelligence research; hence, there is a lack of communication or understanding across academic subfields (of all kinds) from the start.

## 5. What Can Be Done about This? 

*What can intelligence researchers do on their end?* In reference to scholars in the field of intelligence, Hunt (2009, p. 324) noted “We have a communication problem.” Given that the body of evidence supporting intelligence and its importance in life is hard to dispute, Hunt is correct that the field most likely has a communication issue. Intelligence researchers must more effectively communicate their research to subfields beyond their own and to the general public, and ideally, the general public would be more receptive to traditionally “controversial” areas of research such as intelligence, especially in the U.S., and particularly within U.S. education (e.g., Gottfredson 2004; Wai et al. 2018).

Given the sensitive nature of the role of intelligence in education and life contexts, one important step that intelligence researchers could take is to communicate research evidence with the full awareness of the political and ideological forces that are at play within the U.S. education community at both the research and practice levels. For example, in the real world, intelligence is widely employed as an important screening tool for a wide range of jobs (Gottfredson 2004) including in higher education (Posselt 2016), though this is not openly acknowledged as such. Intelligence researchers could potentially be more effective in communicating the importance of intelligence research for education research and policy by working to continually teach others about intelligence research with a focus on how taking evidence into account, much like in health, can more rapidly lead to effective solutions to help children. As Detterman (2016) has pointed out, we may first need to understand intelligence to understand its impacts on education and possibilities in helping kids.

*What can be done in regards to the education community and those who work in schools?* One leverage point that may be useful in getting the concept of intelligence more integrated into the education community and schools is to leverage “school choice,” something many U.S. educators and parents care deeply about (e.g., Ladner 2018). For example, gifted education is connected to high intelligence and people who have kids who are bored or unchallenged (or often bullied) in K-12 traditional public schools would be motivated to find schooling options which would best challenge their kids to develop their talents to the fullest (e.g., Assouline et al. 2015). Interestingly, as Ladner (2018) and Kronholz (2014) point out, in relatively free education markets disproportionate numbers of American parents choose classical academies and similar schools with considerable emphasis on academic content, and where, as Kronholz puts it “teachers are scholars.” Accordingly, over the long term, more school choice might bring more consideration of intelligence into schooling.

Second, pipelines into school leadership prioritize athletic coaching, an area often antithetical to advancement placement and gifted education (Maranto et al. 2018). Acknowledging the concept of intelligence as important to education could over the long-term lead to serious discussions regarding the cognitive capacities of school teachers and leaders. Indeed, one of us (Maranto 2018) has argued for substantially higher teacher pay to attract more cognitively able individuals to the field, coupled with serious accountability systems. A related possibility, hinted at by Stotsky (2015), would be anything bringing additional transparency to the relatively low academic achievement of public educators as measured by tests such as the Praxis, which in part likely measure intelligence. Given the aforementioned public and elite demands for academically talented teachers and school leaders, as expressed in school choice, such transparency could lead to political pressures to reform pipelines into teaching and school leadership. For example, were the U.S. Department of Education to rank states by the mean ACTs and SATs of new entrants to teaching and school leadership, state level officials might feel incentives to reform the human capital pipeline into education. One could also imagine national level teacher and leader certification systems, which would be more transparent than state systems. This transparency could encourage reforms bringing higher levels of human capital in public education.

A third leverage point that may be useful is anything showing solid replicable evidence that intelligence is malleable. To date, such evidence remains unclear (e.g., see Haier 2016), but both the Flynn effect (e.g., Flynn 1984, 1987) and how education may potentially improve intelligence (e.g., Ceci 1991; Ceci and Williams 1997; Ritchie and Tucker-Drob 2018) may have potential. Beyond *g*, some of the literature on specific abilities like spatial ability, has shown some support for the idea of malleability (e.g., Uttal et al. 2013; Stieff and Uttal 2015), though again the evidence remains unclear whether such changes in spatial reasoning will necessarily lead to lasting changes in the ability constructs and long-term predictive validity or if these changes might “fade out” over time (e.g., as they do for interventions targeting cognitive skills: Bailey et al. 2016; Protzko 2015). Whitehurst (2019), for example, has argued that policies targeting the malleability of ability or personality constructs, for example, are unlikely to be fruitful at scale. Of course, because the hope for the malleability of intelligence is so strong, the rapid adoption of ideas such as “brain training” into schools may have brought intelligence into education—just not good evidence or application of intelligence given the enormous lack of evidence supporting brain training (e.g., see Simons et al. 2016). We emphasize ensuring that there is solid replicable evidence for any concept prior to its adoption in schools or interventions (e.g., Asbury and Wai 2019), though, in practice the field of education is quite susceptible to fads with little evidence at all (Hirsch 1996; Ravitch 2000), so new catchy ideas that lack evidence will in all likelihood continue to be adopted in schools.

## 6. Conclusions

As Arvey et al. (1994) wrote in *The Wall Street Journal*, “The research findings [on intelligence] neither dictate nor preclude any particular social policy, because they can never determine our goals. They can, however, help us estimate the likely success and side-effects of pursuing those goals via different means.” We hope that this brief review of history and consideration of the notion of intelligence in education is helpful to both the intelligence research community and also to the education research and policy communities. In particular, we hope this cross-disciplinary collaboration between a political scientist and a psychologist could offer a model for further exploration of these and other issues. The notion of intelligence most certainly should, in our view, be accounted for in education policy and practice. As well, in fact, surveys and focus groups indicate that within the U.S., other western countries, and non-western countries, teachers acknowledge the importance of intelligence (e.g., Castera and Clement 2014; Martschenko 2019). In short, educators in the classroom recognize the importance of intelligence; thus, it should be an important area of study for education researchers. Whether that will happen in the U.S. to a significant degree remains to be seen, though as in health, we believe that taking all the evidence into account will, over the long term, help children in schools. Perhaps the most important issue at hand to figure out is how evidence-based practice can be more successfully adopted in schools. Ignoring the accumulated scientific evidence would seem unethical in medicine (e.g., Upshur 2013), but for whatever reason it remains acceptable in education. Perhaps it is time for the evidence-gap between what we know about intelligence and how education is practiced, to begin to narrow in a meaningful way. Perhaps a closer connection of scientific rigor in education-related intelligence research supplemented by implementation research showing the relevance of considering intelligence in school accompanied by more public communication of high-quality scientific results and continuous cooperation with educational stakeholders might, in the long run, move the needle towards improving schools and education. However, if history is our guide, the challenge to integrate intelligence into U.S. education will very likely remain for some time to come.

## References

[B1-jintelligence-08-00002] Armstrong Elizabeth A., Hamilton Laura T. (2013). Paying for the Party: How College Maintains Inequality.

[B2-jintelligence-08-00002] Arvey Richard D., Bouchard Thomas J., Carroll John B., Cattell Raymond B., Cohen David B., Dawis Rene V., Willerman L. (1994). Mainstream science on intelligence. The Wall Street Journal.

[B3-jintelligence-08-00002] Asbury Kathryn A., Wai Jonathan (2019). Viewing Education Policy through a Genetic Lens. Journal of School Choice.

[B4-jintelligence-08-00002] Assouline Susan, Colangelo Nicholas, VanTassel-Baska Joyce, Lupkowski-Shoplik Anne (2015). A Nation Empowered: Evidence Trumps the Excuses that Hold Back America’s Brightest Students.

[B5-jintelligence-08-00002] Bailey Drew, Duncan Greg J., Odgers Candice L., Yu Winnie (2016). Persistence and fadeout in the impacts of child and adolescent interventions. Journal of Research on Educational Effectiveness.

[B6-jintelligence-08-00002] Bowen Daniel H., Buck Stuart, Deck Cary, Mills Jonathan N., Shuls James V. (2015). Risky business: An analysis of teacher risk preferences. Education Economics.

[B7-jintelligence-08-00002] Burkett Elinor (2001). Another Planet: A Year in the Life of a Suburban High School.

[B8-jintelligence-08-00002] Callahan Raymond E. (1962). Education and the Cult of Efficiency.

[B9-jintelligence-08-00002] Carroll John B. (1997). Psychometrics, intelligence, and public perception. Intelligence.

[B10-jintelligence-08-00002] Castera Jeremy, Clement Pierre (2014). Teachers’ conceptions about the genetic determinism of human behavior: A survey in 23 countries. Science and Education.

[B11-jintelligence-08-00002] Ceci Stephen J. (1991). How much does schooling influence intellectual development and its cognitive components? A reassessment of the evidence. Developmental Psychology.

[B12-jintelligence-08-00002] Ceci Stephen J., Williams Wendy M. (1997). Schooling, intelligence, and income. American Psychologist.

[B13-jintelligence-08-00002] Chubb John E., Moe Terry M. (1990). Politics, Markets, and America’s Schools.

[B14-jintelligence-08-00002] College Board (2014). The 2014 SAT Report on College & Career Readiness. https://research.collegeboard.org/programs/sat/data/archived/cb-seniors-2014.

[B15-jintelligence-08-00002] Demerath Peter (2009). Producing Success: The Culture of Personal Advancement in an American High School.

[B16-jintelligence-08-00002] Detterman Douglas K. (2016). Education and intelligence: Pity the poor teacher because student characteristics are more significant than teachers or schools. Spanish Journal of Psychology.

[B17-jintelligence-08-00002] Flynn James R. (1984). The mean IQ of Americans: Massive gains 1932 to 1978. Psychological Bulletin.

[B18-jintelligence-08-00002] Flynn J. R. (1987). Massive IQ gains in 14 nations: What IQ tests really measure. Psychological Bulletin.

[B19-jintelligence-08-00002] Frey Meredith C., Detterman Douglas K. (2004). Scholastic assessment or g? The relationship between the SAT and general cognitive ability. Psychological Science.

[B20-jintelligence-08-00002] Gastic B., M. Hess Frederick, McShane Q. (2014). Closing the opportunity gap: Preparing the next generation of effective teachers. Teacher Quality 2.0: Toward a New Era in Education Reform.

[B21-jintelligence-08-00002] Gottfredson Linda S. (2004). Schools and the *g* factor. The Wilson Quarterly.

[B22-jintelligence-08-00002] Haier Richard J. (2016). The Neuroscience of Intelligence.

[B23-jintelligence-08-00002] Herrnstein Richard J., Murray Charles (1996). The Bell Curve: Intelligence and Class Structure in American Life.

[B24-jintelligence-08-00002] Hirsch Eric Donald (1996). The Schools We Need and Why We Don’t Have Them.

[B25-jintelligence-08-00002] Hunt Earl (2009). Good news, bad, news, and a fallacy: A review of *Outliers: The Story of Success*. Intelligence.

[B26-jintelligence-08-00002] Ingersoll Richard M. (2003). Who Controls Teachers’ Work?.

[B27-jintelligence-08-00002] Jensen Arthur R. (1969). How much can we boost IQ and scholastic achievement?. Harvard Educational Review.

[B28-jintelligence-08-00002] Jensen Arthur R. (1998). The g Factor: The Science of Mental Ability.

[B29-jintelligence-08-00002] Klyza Christopher M. (1996). Who Controls Public Lands? Mining, Forestry, and Grazing Policies, 1870–1990.

[B30-jintelligence-08-00002] Koedel Cory (2011). Grade Inflation for Education Majors and Low Standards for Teachers: When Everyone Makes the Grade.

[B31-jintelligence-08-00002] Koenig Katherine A., Frey Meredith C., Detterman Douglas K. (2008). ACT and general cognitive ability. Intelligence.

[B32-jintelligence-08-00002] Kramer Rita (1991). School Follies.

[B33-jintelligence-08-00002] Kronholz June (2014). High Scores at BASIS Charter Schools. Education Next.

[B34-jintelligence-08-00002] Labaree David F. (2003). The Trouble with Ed. Schools.

[B35-jintelligence-08-00002] Labaree David F., Ravitch Diane (2004). The Ed school’s romance with progressivism. Brookings Paper on Education Policy.

[B36-jintelligence-08-00002] Ladner Matthew, Greene Jay P., McShane Michael Q. (2018). No excuses charter schools: The good, the bad, and the over-prescribed. Failure Up Close: What Happens, Why it Happens, and What We Can Learn From it.

[B37-jintelligence-08-00002] Levine Arthur (2006). Educating School Teachers.

[B38-jintelligence-08-00002] Loveless Tom (1999). The Tracking Wars: State Reform Meets School Policy.

[B39-jintelligence-08-00002] Lucas Christopher J. (1999). Teacher Education in America.

[B40-jintelligence-08-00002] Maeroff Gene I. (2010). School Boards in America: A Flawed Exercise in Democracy.

[B41-jintelligence-08-00002] Maranto Robert, Maranto Robert, Milliman Scott, Hess Frederick, Gresham April (2001). The death of one best way: Charter schools as reinventing government. School Choice in the Real World: Lessons from Arizona Charter Schools.

[B42-jintelligence-08-00002] Maranto Robert (2015). Why don’t schools teach poetry?. Academic Questions.

[B43-jintelligence-08-00002] Maranto Robert (2018). Pay teachers more—But make sure they earn it. Wall Street Journal.

[B44-jintelligence-08-00002] Maranto Robert, McShane Michael Q. (2012). President Obama and Education Reform: The Personal and the Political.

[B45-jintelligence-08-00002] Maranto Robert, Ritter Gary, Levine Arthur (2010). The Future of Ed. schools: Five lessons from business schools. Education Week.

[B46-jintelligence-08-00002] Maranto Robert, Carroll Kristen, Cheng Albert, P. Teodoro Manuel (2018). Boys will be superintendents: School leadership as a gendered profession. Phi Delta Kappan.

[B47-jintelligence-08-00002] Martschenko Daphne (2019). DNA dreams’: Teacher perspectives on the role and relevance of genetics for education. Research in Education.

[B48-jintelligence-08-00002] Mehta Jal (2013). The Allure of Order.

[B49-jintelligence-08-00002] Mehta Jal, Teles Steven, M. Hess Frederick, Q. McShane Michael (2014). Professionalism 2.0: The case for plural professionalism. Teacher Quality 2.0: Toward a New Era in Education Reform.

[B50-jintelligence-08-00002] Moe Terry (2011). Special Interest: Teachers Unions and America’s Public Schools.

[B51-jintelligence-08-00002] Ouchi William G. (2009). The Secret of TSL.

[B52-jintelligence-08-00002] Perrow Charles (1979). Complex Organizations: A Critical Essay.

[B53-jintelligence-08-00002] Posselt Julie R. (2016). Inside Graduate Admissions: Merit, Diversity, and Faculty Gatekeeping.

[B54-jintelligence-08-00002] Powell Arthur G., Farrar Eleanor, Cohen David K. (1985). The Shopping Mall High School: Winners and Losers in the Educational Marketplace.

[B55-jintelligence-08-00002] Protzko John (2015). The environment in raising early intelligence: A meta-analysis of the fadeout effect. Intelligence.

[B56-jintelligence-08-00002] Ravitch Diane (2000). Left Back: A Century of Failed School Reforms.

[B57-jintelligence-08-00002] Ritchie Stuart J., Tucker-Drob Elliot M. (2018). How much does education improve intelligence? A meta-analysis. Psychological Science.

[B58-jintelligence-08-00002] Rousmaniere Kate (2013). The Principal’s Office: A Social History of the American School Principal.

[B59-jintelligence-08-00002] Schmidt Frank L., Hunter John E. (1998). The validity and utility of selection methods in personnel psychology: Practical and theoretical implications of 85 years of research findings. Psychological Bulletin.

[B60-jintelligence-08-00002] Schmidt Frank L., E. Hunter John (2004). General mental ability in the world of work: Occupational attainment and job performance. Journal of Personality and Social Psychology.

[B61-jintelligence-08-00002] Shuls James V., Trivitt Julie R. (2015). Teacher qualifications and productivity in secondary schools. Journal of School Choice.

[B62-jintelligence-08-00002] Simons Daniel J., Boot Walter R., Charness Neil, Gathercole Susan E., Chabris Christopher F., Hambrick David Z., Stine-Morrow Elizabeth A. L. (2016). Do “brain training” programs work?. Psychological Science in the Public Interest.

[B63-jintelligence-08-00002] Stieff Mike, Uttal David H. (2015). How much can spatial training improve STEM achievement?. Educational Psychology Review.

[B64-jintelligence-08-00002] Stotsky Sandra (2015). An Empty Curriculum: The Need to Reform Teacher Licensing.

[B65-jintelligence-08-00002] Thernstrom Abigail, Thernstrom Stephan (2003). No Excuses: Closing the Racial Gap in Learning.

[B66-jintelligence-08-00002] Tyack David B. (1974). The One Best System.

[B67-jintelligence-08-00002] Tyack David, Cuban Larry (1995). Tinkering Toward Utopia: A Century of Public School Reform.

[B68-jintelligence-08-00002] Upshur Ross E. G. (2013). A call to integrate ethics and evidence-based medicine. AMA Journal of Ethics.

[B69-jintelligence-08-00002] Uttal David H., Meadow Nathaniel G., Tipton Elizabeth, Hand Linda L., Alden Alison R., Warren Christopher, Newcombe Nora (2013). The malleability of spatial skills: A meta-analysis of training studies. Psychological Bulletin.

[B70-jintelligence-08-00002] Wai Jonathan (2015). Your college major is a pretty good indication of how smart you are. Quartz.

[B71-jintelligence-08-00002] Wai Jonathan, Lubinski David, Benbow Camilla P. (2009). Spatial ability and STEM domains: Aligning over fifty years of cumulative psychological knowledge solidifies its importance. Journal of Educational Psychology.

[B72-jintelligence-08-00002] Wai Jonathan, Brown Matt I., Chabris Christopher F. (2018). Using standardized test scores to include general cognitive ability in education research and policy. Journal of Intelligence.

[B73-jintelligence-08-00002] Whitehurst Grover J. (2019). A prevalence of “policy-based evidence-making”. Education Next.

[B74-jintelligence-08-00002] Wolfle Dael, Oxtoby Toby (1952). Distributions of ability of students specializing in different fields. Science.

